# Proteomic Analysis of Extracellular Vesicles From *Fasciola hepatica* Hatching Eggs and Juveniles in Culture

**DOI:** 10.3389/fcimb.2022.903602

**Published:** 2022-06-03

**Authors:** María Trelis, Christian M. Sánchez-López, Liz F. Sánchez-Palencia, Victor Ramírez-Toledo, Antonio Marcilla, Dolores Bernal

**Affiliations:** ^1^ Área de Parasitología, Departament de Farmàcia i Tecnologia Farmacèutica i Parasitologia, Universitat de València, Valencia, Spain; ^2^ Joint Research Unit on Endocrinology, Nutrition and Clinical Dietetics, Health Research Institute La Fe, Universitat de Valencia, Valencia, Spain; ^3^ Veterinari de Salut Pública, Centre de Salut Pública de Manises, Valencia, Spain; ^4^ Departament de Bioquímica i Biologia Molecular, Facultat de Ciències Biològiques, Universitat de València, Valencia, Spain

**Keywords:** extracellular vesicles, helminth proteomics, NEJ, *Fasciola hepatica*, eggs, miracidia

## Abstract

The identification of extracellular vesicles (EVs) in *Fasciola hepatica* has provided a new way to understand parasite-host communication. Most of the studies on EVs have focused on the adult stage of *F. hepatica*, but recently, the presence of EVs from different developmental stages has been reported. To better understand the potential role of EVs in the biology of the parasite and in the infection process, the protein cargo of EVs from embryonated eggs and newly-excysted juvenile (NEJs) flukes cultured up to 28 days, has been analyzed. EVs were isolated by size exclusion chromatography and evaluated by nanoparticle tracking analysis and transmission electron microscopy. LC-MS/MS proteomic analysis of EVs revealed the presence of 23 different proteins from embryonated egg-derived EVs and 29 different proteins from NEJ-derived EVs. Most of the identified proteins had been previously described in EVs from *F. hepatica* adults, including cytoskeletal proteins, glycolytic enzymes, stress-related proteins and tetraspanins. Nevertheless, EVs from hatching eggs and NEJs exhibited qualitative differences in composition, when compared to EVs form adults, including the absence of cathepsin cysteine peptidases. The differential content of the EVs released by the different developmental stages of the parasite reflect the intense activity of NEJs at this early stage, with several proteins involved in membrane traffic and cell physiology. This new set of identified proteins could help to understand key metabolic, biochemical and molecular mechanisms mediated by EVs that take place upon egg hatching and after parasite excystment.

## Introduction

Fascioliasis, an infection caused by trematodes of the genus *Fasciola*, is a major neglected zoonotic disease that infects humans and ruminant species worldwide. The first description of extracellular vesicles (EVs) in the excretory-secretory products (ESP) of *Fasciola hepatica* adult worms in 2012 provided a new way to understand helminth-host communication (Marcilla et al., 2012). Since then, an increasing number of studies describing the presence of EVs in different parasitic helminths species have been published (reviewed in Marcilla et al., 2014; Coakley et al., 2016; Tritten and Geary, 2018; Mardahl et al., 2019; Hoffmann et al., 2020; Sotillo et al., 2020). Helminths are an excellent example of parasites with the ability to modulate the host immune system through EVs, preventing parasite expulsion (Montaner et al., 2014; Zakeri et al., 2018; Sánchez-López et al., 2021).

EVs from *F. hepatica* (*Fh*EVs) adults have been characterized, and the protein and miRNA content has been determined, revealing the involvement of these structures and their cargo in the communication with the host, their role in the establishment of the infection and their potential in the diagnosis and control of parasitic infections (Marcilla et al., 2012; Fromm et al., 2015). A high degree of morphological variability in *Fh*EVs, purified by size exclusion chromatography (SEC), has been recently reported (Sánchez-López et al., 2020), which suggests the existence of heterogeneous subpopulations with different functions and biochemistry. In this context, two subpopulations of EVs from *F. hepatica* adults, which differ according to size, cargo molecules and potential site of release from the parasite have been characterized. The large 15K *Fh*EVs (termed microvesicles) are enriched for the 37 kDa inactive zymogen of the cathepsin L1, a digestive peptidase that can be activated by a low pH. In contrast, the small 120K *Fh*EVs (termed small exosome-like EVs) contain a number of exosomal markers including Hsp70, ALIX and tetraspanins, as well as peptidases, which are highly represented (Cwiklinski et al., 2015). This study suggests that the cargo molecules packaged within the EVs are highly specific, likely to facilitate parasite migration through host tissues and to evade host immune attack.

Mammalians and Lymnaeid snails are the definitive and intermediate hosts during the life cycle of *F. hepatica*. Definitive hosts, including humans become infected upon metacercariae ingestion. Once inside the host, Newly Excysted Juveniles (NEJs) are released after the metacercarial cyst wall is digested in the small intestine of the host within an hour. The NEJs then penetrate the host intestine wall, and in about 2 h, they are found in the abdominal cavity. Within 4-6 days, they reach the liver by crossing the peritoneal space. For the next 5 to 6 weeks, extensive hemorrhaging and fibrosis can be caused when the young flukes migrate through the liver parenchyma. After approximately 7 weeks, the flukes reach the bile ducts. There, they become sexually mature and begin laying eggs (Andrews, 1999). Although, the migration of *F. hepatica* in the liver parenchyma is considered to be controversial, accrued data regarding the secretion of the cathepsin L and B cysteine peptidases add support to the currently accepted life cycle described above (Moazeni and Ahmadi, 2016). It is still unclear whether very small juvenile flukes enter the bile ducts upon arrival in the liver parenchyma or if they reach the hepatic bile ducts through the ductus choleduchus, where they mature. Support for the hypothesis that the flukes migrate to the liver through the abdominal cavity comes from the observation that NEJs do not survive in bile containing solutions. However, a direct route through the main bile duct that starts at the small intestine cannot be formally discarded (González-Miguel et al., 2021).

Recently, the secretion of EVs from different developmental stages of *F. hepatica*, including embryonated eggs, and NEJs cultured up to 24 days has been described (Sánchez-López et al., 2020). The aim of the present study is to analyze the protein cargo of these *Fh*EVs in order to infer their role in the early stages of the parasite life cycle, and to identify EV proteins with the potential to interact with the host immune system, preventing parasite expulsion. These proteins could be of potential interest for developing methods for disease control.

## Materials and Methods

### Eggs and Newly Excysted Juveniles From *F. hepatica*

Adults of *F. hepatica* were obtained from infected cattle livers from local slaughterhouses. The eggs from *F. hepatica* were obtained in the laboratory as previously described (Sánchez-López et al., 2020). Briefly, 24 reproductively mature adults were maintained at 37°C for 5h at concentrations of 2 individuals/mL in RPMI-1640 culture medium (Life Technologies), containing 100 U penicillin, 100 mg/mL streptomycin (all from Sigma), and a cocktail of protease inhibitors (cOmplete™, EDTA-free, Roche). To collect the eggs actively released by adults, culture media was centrifuged at 750 xg for 10 min, and then eggs were washed thoroughly with 0.01 M saline phosphate buffer (PBS).

*F. hepatica* metacercariae were obtained from Ridgeway Research (St Briavels, Gloucestershire, UK), and stored at 4°C until required. 1000 metacercariae were excysted as previously described (McVeigh et al., 2014; Sánchez-López et al., 2020). Metacercariae were physically popped from their outer wall using a razor blade, and/or by incubation in a solution containing 1% pepsin and 4 mM HCl, for 90 min at 37°C. Metacercariae were excysted by incubation in 0.6% sodium bicarbonate, 0.45% sodium chloride, 0.4% sodium tauroglycocholate, 0.025 M HCl, 0.4% L-cysteine. NEJs were transferred individually to RPMI-1640 (Life Technologies), in which they were maintained at 37°C for a maximum of 1 h until transferred to soaking media. 60% of NEJs were alive after the first day.

### Culture of NEJs and Egg Hatching of *F. hepatica*

NEJs were maintained in groups of 10-15 per well in 24-well plates, in 500 µL of RPMI-1640 culture medium (Life Technologies) supplemented with 50% chicken serum (Sigma-Aldrich) and 100 U/mL of penicillin, 0.1 mg/mL of streptomycin, and 0.25 µg/mL of amphotericin B (all from Sigma-Aldrich). NEJs were maintained in a humidified incubator at 37°C with 5% CO_2_ up to 28 days, and were visualized every day under the microscope, assessing their survival as described (Sánchez-López et al., 2020). Every 3 days, NEJs were washed twice with RPMI-1640, to prevent contamination with chicken serum EVs, transferred individually to a new well, and incubated in the same medium for 4 h. After the incubation period, the parasite culture media was collected and centrifuged, first at 3,000 xg/10 min at 4°C, and the collected supernatant was then centrifuged at 15,000 xg for 30 min at 4°C. These supernatants contained the ESP from the *F. hepatica* juveniles. In all cases, parasites were alive after incubations. After obtaining the ESP, juveniles were kept in culture in the RPMI-1640 culture medium containing 50% chicken serum as described above.

All the ESP obtained from *F. hepatica* juveniles were collected as a pool since the amount of EVs obtained from this developmental stage of the parasite was very limited.

To obtain the ESP from mature eggs, they were first embryonated after their incubation in mineral water at 24°C for 28 days in darkness and then placed under light for 2 h to stimulate the hatching of the miracidia. The percentage of hatched eggs was higher than 90%. When the miracidia lost mobility, the ESP was recovered and centrifuged at 4°C, first at 3000 xg/10 min, and the collected supernatant was centrifuged at 15,000 xg for 30 min.

### Isolation of EVs by Size Exclusion Chromatography and Characterization by Nanoparticle Tracking Analysis and TEM

The ESP from NEJs and hatching eggs were filtered through a 0.22 µm filter (Thermo Fisher Scientific), and concentrated using Amicon Ultra-4 filter devices (Merck Millipore) at 3,200 xg for 20 min at 4°C. This step was repeated with the same filter to reach a final volume of 50 µL for both juvenile and egg samples. Prior to EV isolation, columns containing 1 mL of stacked Sepharose-CL2B (Sigma-Aldrich) were equilibrated by washing them with 3 mL of PBS previously filtered using 0.22 µm filters. Samples were passed through the columns, and filtered PBS was used as the elution buffer. 50 µL of concentrated ESP was loaded onto the column, and a total of 12 70 µL fractions were collected. EVs from NEJs and eggs eluted in fractions 4–7, which were pooled (Sánchez-López et al., 2020).

Size distribution of particles was determined by NTA in a NanoSight LM10 (Malvern Instrument Ltd, Malvern, UK), using a 405 nm laser and a sCMOS camera. Data were analyzed with the NTA software version 3.3 (Dev Build 3.3.104), as previously described (Sánchez-López et al., 2020). Samples were diluted with filtered PBS to reach the concentration recommended by the manufacturer (20-120 particles/frame).

To verify the presence of EVs, aliquots of purified EVs were processed for negative staining with 2% uranyl acetate as previously described (Théry et al., 2006; Sánchez-López et al., 2020) and analyzed at the Microscopy Service of the Prince Felipe Research Centre of Valencia, Spain (CIPF). EVs were visualized with a Jeol JEM1010 transmission electron microscope at 80 kV (Servicio Central de Soporte a la Investigación Experimental (SCSIE), Universitat de València, Spain).

### Proteomic Analysis

Before sample processing, a 1:1 combination of two different types of carboxylate-modified magnetic particles (Thermo Scientific Sera-Mag SpeedBeads™) were used to remove contaminants. Beads were rinsed three times in 1mL of water, placing the beads on a magnetics rack (MagneSphere, Promega, cat. no. Z5342) and removing the supernatant each time. Then, 500 μL of water were added to the beads for storage at 4°C as a 20 μg/μL stock concentration of beads.

To prepare the samples, purified extracellular vesicles were dried and dissolved in 30 μL of 50 mM ammonium bicarbonate (ABC). 4 μL of beads were added to each sample and then, immediately, acetonitrile (ACN) was added to a final concentration of 70% (v/v). The sample/bead mixture was briefly vortex, incubated for 20 min at RT and placed on a magnetic rack for 2 min, discarding the supernatant. The beads were then washed twice with 200 μL of 70% EtOH, and once with 180 μL of ACN, incubating the beads for 30 s on the magnetic rack and discarding the supernatant each time, allowing them to dry for 2 min to ensure complete removal of ACN. For elution, the washed beads were resuspended in 45 μL of 50 mM ABC buffer, and treated overnight with 500 ng trypsin (Promega) in a final volume of 50 μL at 37°C. Digestions were stopped with 60 μL of Trifluoroacetic acid (TFA) (Sigma-Aldrich) 10% (final pH=1). The tubes were centrifuged for 1 min to pellet the beads, and the supernatants containing peptides were collected using a plate magnet. Peptide mixtures were concentrated by speed vacuum to 10 μL.

The final peptide mixtures were analyzed by liquid chromatography and tandem mass spectrometry (LC–MS/MS) in a nanoESI qQTOF 6600plus TripleTOF (Sciex) mass spectrometer coupled to a nanoLC Ekspert nanoLC-425 (Eksigent). First, 5 µL of the tryptic peptides were loaded to a trap column (3 µm C18-CL, 350 μm x 0.5mm; Eksigent), and desalted with 0.1% TFA at 5 µL/min for 5 min. The cleaned peptides were then eluted onto an analytical column (3µm C18-CL 120 Ᾰ, 0.075 x 150 mm; Eksigent) as described previously (Cwiklinski et al., 2015). The final elution of the peptides was carried out with a linear gradient of 15 to 40% of solution B (ACN, 0.1% FA) in A (0.1% FA) for 60 min at a flow rate of 300 nL/min. The eluted peptides were ionized in an Optiflow Source < 1 µL Nano (Sciex) applying 3.0 kV to the spray emitter at 175°C. Acquisition was performed in a data-dependent mode (DDA) with survey MS1 scans from 350 to 1400 m/z during 250 ms. The MS2 experiments were acquired from 100 to 1500 m/z for 25 ms in ‘high sensitivity’ mode with the quadrupole resolution set to ‘LOW’. The proteomic analysis was performed in the proteomics facility of the SCSIE, Universitat de València.

### Database Search

Database search in protein databases was performed using the ProteinPilot search engine v5.0 (ABSciex). ProteinPilot default parameters were used to generate peak lists directly from 6600 plus TripleTOF *wiff* files. The Paragon algorithm (Shilov et al., 2007) of ProteinPilot v 5.0 was used to search the Uniprot_trematoda database (200604, 362615 proteins). The parameters used were: Trypsin specificity, IAM cys-alkylation, no taxonomy restriction, and the search effort set to rapid with FDR analysis. The protein grouping was done using the Pro-group algorithm. The mass spectrometry proteomics data have been deposited to the ProteomeXchange Consortium *via* the PRIDE (Perez-Riverol et al., 2022) partner repository with the dataset identifier PXD033086.

## Results

The low amount of EVs, and therefore of proteins, requires robust protocols and reagents to efficiently handle and enrich these molecules prior to analysis with mass spectrometry. A method based on the single‐pot solid‐phase‐enhanced sample preparation (SP3) was used for the automated sample preparation pipeline for low‐input proteomics (Moggridge et al., 2018; Müller et al., 2020).

### Identification of Protein Cargo in EVs From *F. hepatica* Eggs/Miracidia

**Figure 1:**
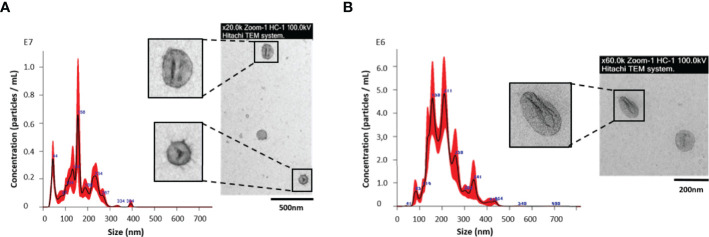
Nanoparticle tracking analysis (NTA) and transmission electron microscopy (TEM) of extracellular vesicles (EVs) from **(A)** *Fasciola hepatica* eggs/miracidia; and **(B)** *F. hepatica* NEJs, cultured up to 28 days, and collected as a pool. Each aliquot of EVs from NEJs and eggs, eluted in fractions 4–7 during SEC isolation, was analyzed by NTA in a NanoSight LM10 using samples diluted 1:50 and 1:80 respectively, to determine the size distribution and concentration. The insets show an image of a representative sample of each preparation processed for negative staining with 2% uranyl acetate as described in M&M and visualized with a Jeol JEM1010 transmission electron microscope at 80 kV.

The identification of protein cargo from egg/miracidium-derived EVs may be determinant for the study of the interaction *in vivo* with the intermediate host. Eggs of *F. hepatica* are shed in the feces of infected mammals. Following a period of maturation, the ciliated miracidium hatches out of the egg until reaching the snail intermediate host. To analyze the protein cargo of the *Fh*EVs from experimentally-embryonated eggs/miracidia, eggs were first placed under light to stimulate hatching of the miracidia, as described above. When the miracidia lost their mobility (indicating they were losing viability), the ESP was recovered and the EVs were purified by SEC. To verify the presence of EVs and to characterize them (size and morphology), both NTA and TEM were used. NTA analyses revealed the presence of vesicles of 158.9 +/- 9.4 nm as a median, and typical round shape morphology (**Figure 1**), confirming previous observations from various developmental stages including embryonated eggs and NEJs (Sánchez-López et al., 2020).

For proteomic analysis, a total of an estimated number of 7.56x10^9^ vesicles were used, and a sample preparation for low-input proteomics was performed using two different types of carboxylate-modified magnetic particles. The proteomic analysis employing LC-MS/MS identified 23 different proteins from embryonated egg/miracidium-derived EVs (**Table 1**).

**Table 1:** Identification of proteins within the EVs of *F. hepatica* eggs/miracidia isolated by SEC and analyzed by LC-MS/MS.

Accession	Description	Species	Total	% Cov	Peptides (95%)
tr|A0A4E0RII0	Uncharacterized protein	*Fasciola hepatica*	16,15	23,72	19
tr|A0A4E0S3Z0	Uncharacterized protein	*Fasciola hepatica*	11,16	8,47	6
tr|A0A2H1CEU3	Phosphoenolpyruvate carboxykinase	*Fasciola hepatica*	6,96	19,12	3
tr|Q06A71	Glutathione transferase sigma class	*Fasciola hepatica*	4,24	20,38	2
tr|A0A504YCL4	Glyceraldehyde-3-phosphate dehydrogenase	*Fasciola gigantica*	2,82	9,20	1
tr|Q71TT8	Actin	*Schistosoma japonicum*	6,62	19,95	3
tr|A0A4E0S3V8	Annexin	*Fasciola hepatica*	6,63	23,03	3
tr|A0A4E0R1A8	Tetraspanin	*Fasciola hepatica*	6,53	19,11	3
tr|A0A4E0RTX9	Tetraspanin-1	*Fasciola hepatica*	5,07	21,62	5
tr|A0A4E0R873	Tetraspanin-6	*Fasciola hepatica*	1,27	8,33	1
tr|A0A504Z380	Tubulin polymerization-promoting protein family member 2	*Fasciola gigantica*	5,36	20,79	3
tr|A0A504X7T8	Putative tegumental protein	*Fasciola gigantica*	2,2	9,19	1
tr|A0A4E0RYW8	Glioma pathogenesis protein 1	*Fasciola hepatica*	5,01	30,40	2
tr|A0A183L0R8	Uncharacterized protein	*Schistosoma curassoni*	4,86	2,13	5
tr|Q9U1G7	Thioredoxin	*Fasciola hepatica*	3,73	29,81	2
tr|A0A504YEZ4	CUB domain-containing protein	*Fasciola gigantica*	3,43	12,46	2
tr|A0A504YMB3	C-type lectin domain-containing protein	*Fasciola gigantica*	2,97	13,43	1
tr|V9LL52	Ubiquitin	*Clonorchis sinensis*	2,92	33,16	2
tr|A0A4E0RJT9	Calpain-B	*Fasciola hepatica*	2,09	6,75	0
tr|A0A2H1CVV7	EF hand	*Fasciola hepatica*	2,01	36,23	3
tr|A0A504YBK9	Uncharacterized protein	*Fasciola gigantica*	1,95	5,16	1
tr|A0A504YEI6	UPAR/Ly6 domain-containing protein	*Fasciola gigantica*	1,7	16,47	2
tr|A0A504Z4R8	Uncharacterized protein	*Fasciola gigantica*	5,01	5,49	3

**Figure 2:**
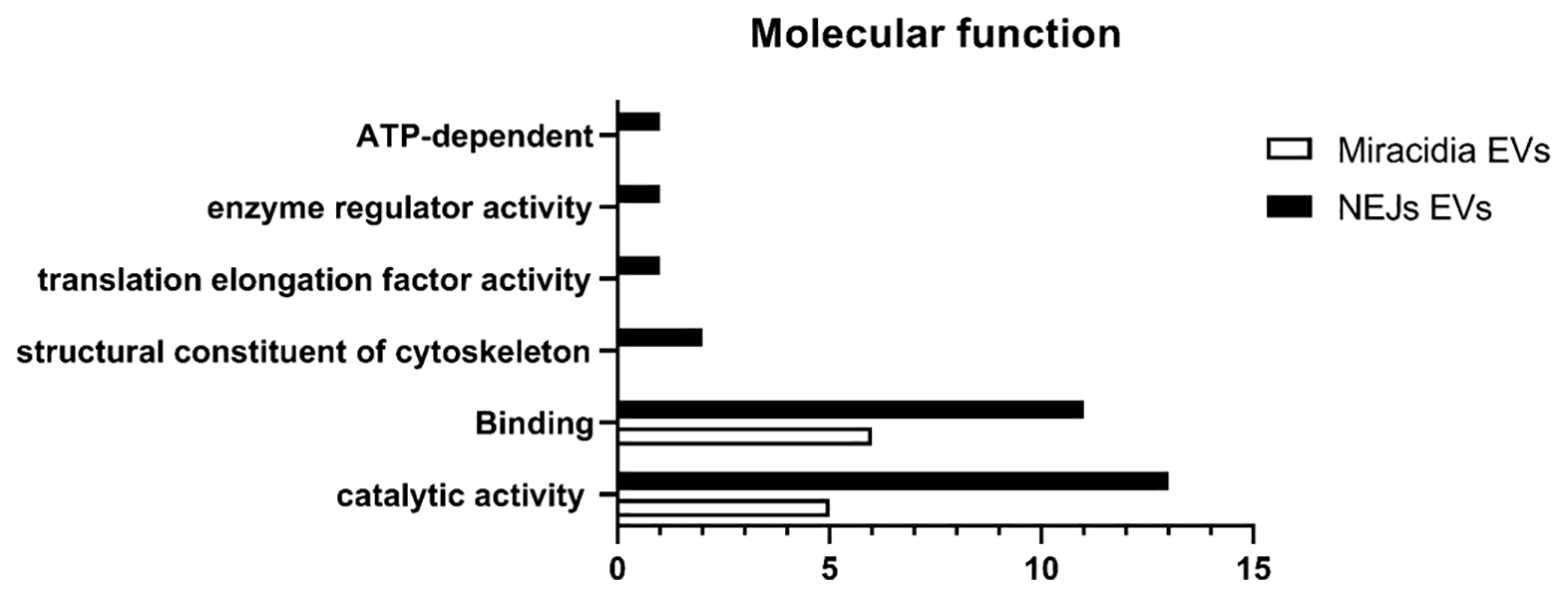
Number of identified proteins in the proteome of *F. hepatica* NEJs and Eggs/Miracidia distributed according to the molecular functions (Gene Ontology, GO, level 2).

The distribution of the molecular functions of proteins identified in eggs/miracidia, according to Gene Ontology (GO) (using the PANTHER-GO system (Version 16.0; http://www.pantherdb.org/), is summarized in **Figure 2**. Most proteins were associated with binding and catalytic activities. The GO term for binding refers to “The selective, non-covalent, often stoichiometric, interaction of a molecule with one or more specific sites on another molecule”. The identified proteins included structural and cytoskeletal proteins such as actin, tubulin and tegumental proteins, as well as proteins involved in exosome biogenesis and/or vesicle trafficking, such as two annexins and three tetraspanins. Among the proteins with catalytic activity, phosphoenolpyruvate carboxykinase (PEPCK), glutathione S-transferase, and the glycolytic enzyme glyceraldehyde-3-phosphate dehydrogenase (GAPDH) were identified. GAPDH is a metabolic enzyme typically found as a component of EVs. Other identified proteins included thioredoxin (involved in defense against immune effector cells), ubiquitin, and calpain.

Interestingly, we have recovered multiple peptides from several uncharacterized proteins, possibly indicating that they were among the most abundant proteins in the EV from hatching eggs. The more abundant protein (accession number A0A4E0RII0) is a protein of 763 amino acids that is predicted to contain a transmembrane domain. Another integral component of membranes is a protein identified in *Schistosoma curassoni*, containing 1032 amino acids (A0A183L0R8). The proteins with accession numbers A0A4E0S3Z0 and A0A504Z4R8 are large glycoproteins of 3662 and 2987 amino acids respectively; the first one shares 50% identity with uncharacterized proteins of *Echinostoma caproni* and *Fasciola gigantica*. Further studies will be required to characterize these proteins and determine their function in these extracellular vesicles.

### Identification of Protein Cargo in EVs From *F. hepatica* NEJs

Recently, we have reported the production and characterization of EVs by NEJs in culture (Sánchez-López et al., 2020). With the aim of identifying proteins involved in the initial steps of *F. hepatica* infection that may be useful in disease control and/or prevention, we investigated the proteins contained in the NEJ-derived EVs. This stage represents the first contact in the host-parasite interface. The low number of released particles by the NEJs, as well as the difficulty of handling the juveniles in culture has impeded the proteomic analysis of EVs over a time-course in culture. To partially overcome these difficulties, and to biochemically characterize the EV protein content in this developmental stage, NEJs were cultured for up to 28 days after excystment from metacercarie, in order to collect an enriched pooled ESP. Every three days, the NEJ culture media was collected and stored at -80°C. The EVs from the pooled samples were purified by SEC as described above. When analyzed by NTA, 3.456 ± 0.72x10^10^ vesicles/mL eluted in fractions 4-7, with an estimated size of 199.6 ± 5.7 nm. The analysis by TEM showed a lower estimated size, with medians of approximately 100 nm (**Figure 1**), as we previously reported (Sánchez-López et al., 2020).

The proteomic analysis, using a total number of 1.21x10^10^ EVs revealed 29 different proteins (**Table 2**). The proteins identified belong to the categories of binding-related molecules, proteins with catalytic activity and proteins with structural functions, as well as proteins involved in vesicular trafficking and formation of vesicles in the secretory and endocytic pathways.

**Table 2:** Identification of proteins within the EVs of *F. hepatica* NEJs in culture isolated by SEC and analyzed by LC-MS/MS.

Accession	Name	Species	emPAI	% Cov	Peptides (95%)
tr|A0A419PN70	Actin-2	*Clonorchis sinensis*	18,41	54,72	18
tr|A0A2H1C8M6	Tubulin alpha chain	*Fasciola hepatica*	16,32	43,58	10
tr|A0A2H1CWM3	Tubulin beta chain	*Fasciola hepatica*	10,26	18,82	6
tr|A0A4E0RTD4	Allergen tropomyosin	*Fasciola hepatica*	2	2,65	1
tr|B1NI98	Heat shock protein Hsp70	*Fasciola hepatica*	5,71	11,01	3
tr|A0A2H1BZF7	Heat shock protein Hsp90	*Fasciola hepatica*	2,9	3,80	1
tr|Q94747	Elongation factor 1-alpha	*Schistosoma mansoni*	2,76	12,26	2
tr|A0A4E0R3E5	Transitional endoplasmic reticulum ATPase	*Fasciola hepatica*	2,54	3,74	1
tr|A0A2H1BWY6	Glyceraldehyde-3-phosphate dehydrogenase	*Fasciola hepatica*	2,19	8,31	1
tr|B2LXU1	Enolase	*Schistosoma bovis*	2,06	9,68	1
tr|A0A2H1CRV9	Pyruvate kinase	*Fasciola hepatica*	2	5,25	1
tr|S4UI50	Triosephosphate isomerase	*Fasciola hepatica*	2	5,93	1
tr|A0A4E0S3H1	Phosphodiesterase-nucleotide pyrophosphatase	*Fasciola hepatica*	2	6,59	1
tr|A0A4Z2CYU8	Dynein beta chain, ciliary	*Schistosoma japonicum*	2	2	1
tr|A0A074Z437	Ras-GEF domain-containing protein (Fragment)	*Opisthorchis viverrini*	2,01	1,76	2
tr|A0A2H1C3P3	14-3-3 protein	*Fasciola hepatica*	2	4,08	1
tr|Q86G46	Ubiquitin (Fragment)	*Schistosoma japonicum*	2	34,12	1
tr|A0A419QA49	BRCT domain-containing protein	*Clonorchis sinensis*	2	1,10	1
tr|A0A4E0RNR9	Small GTP-binding protein Rab1	*Fasciola hepatica*	2	5,53	1
tr|A0A4E0RWP3	Chromobox protein 1 (Heterochromatin protein 1 beta)	*Fasciola hepatica*	2	6,48	1
tr|A0A4E0QYB1	ADP-ribosylation factor	*Fasciola hepatica*	2	9,94	1
tr|A0A4E0S334	SEA domain-containing protein	*Fasciola hepatica*	2	8,05	1
tr|A0A4E0R8T9	Uncharacterized protein	*Fasciola hepatica*	2	10,95	1
tr|A0A2H1C1T9	Uncharacterized protein	*Fasciola hepatica*	2	5,59	1
tr|A0A2H1C1S8	CUB domain protein	*Fasciola hepatica*	2	4,26	2
tr|A0A183W218	Peptidase S1 domain-containing protein	*Trichobilharzia regenti*	2	7,03	1
tr|A0A183B1D0	Uncharacterized protein	*Echinostoma caproni*	2	9,66	1
tr|G7YEZ9	Roundabout homolog 2	*Clonorchis sinensis*	1,8	0,83	1
tr|A0A183JZS7	Uncharacterized protein	*Schistosoma curassoni*	2	44,59	1

The group of proteins corresponding to structural/cytoskeletal function included actin-2, tubulins, tropomyosin and dynein beta chain. Five metabolic enzymes were also identified, including the glycolytic enzymes GAPDH, enolase, pyruvate kinase and triosephosphate isomerase (TIM), and the phosphodiesterase-nucleotide pyrophosphatase. The chaperones Hsp70 (a reported exosomal marker) and Hsp90 were also identified.

Several molecules associated with membrane structure included the Ras-GEF domain containing protein, small GTP-binding protein Rab1, a CUB domain protein, ADP-ribosylation factor and roundabout homolog 2. The Ras superfamily is formed by small monomeric GTPases with more than 170 members. CUB domains are widely occurring structural motifs, found almost exclusively in secreted proteins and in a few plasma membrane-associated proteins.

In addition, other proteins identified included the elongation factor 1-alpha, a 14-3-3 isoform, ubiquitin, a SEA (sea-urchin sperm protein, enterokinase and agrin) domain-containing protein, the transitional endoplasmic reticulum ATPase, and the chromobox protein 1 and a peptidase S1 protein. The four uncharacterized proteins identified in NEJ-EVs are small proteins/peptides with no other homologies with other organisms. The protein/peptide with accession number A0A183JZS7 contains 74 amino acids of which 50% are Ser residues.

## Discussion

Recently, we have reported the capacity of different developmental stages of *F. hepatica*, including eggs (with miracidia) and juvenile flukes (NEJ), to secrete EVs (Sánchez-López et al., 2020). To shed light on the biology of the parasite and the role of EVs in parasite-host communication in the initial steps of the infection by *F. hepatica*, as well as to identify potential candidates to control the disease, we have analyzed the proteomic composition of *F. hepatica* EVs derived from hatching eggs and from cultured immature juveniles.

EV purification was challenging due to the small amount of EVs secreted by eggs and their miracidia, as well as by NEJs. It was necessary to implement a protocol for high-efficiency recovery of functional EVs with enough quality to permit proteomic analysis. EVs were purified by SEC, which is a good technique to separate protein aggregates and lipoproteins from EV samples (De Menezes-Neto et al., 2015). In addition, recent reports have shown that SEC-mediated isolation produces increased EV purity compared to other isolation techniques, such as differential centrifugation (Davis et al., 2019; Sánchez-López et al., 2020).

To date, there are a limited number of proteomic analyses of helminth eggs from trematodes, including the study on *Schistosoma japonicum* eggs and egg-derived ESP proteins (De Marco Verissimo et al., 2019). In that study, 95 proteins were found exclusively in *S. japonicum* ESP. This may suggest that these proteins may be recognized by host immune system effector elements and that they may use several pathways to stimulate the innate and adaptive immune system. More reports have been published on proteomics of juvenile stages from trematodes, including *F. hepatica*, where they migrate through the liver tissue causing the clinical manifestations associated with acute animal and human fasciolosis (Mas-Coma et al., 2005). Among these reports, a proteomic approach identified a set of proteins enriched at the host-parasite interface from NEJ of *F. hepatica* (Hernández-González et al., 2010). In that study, the proteins generated by NEJs were analyzed by LC–MS/MS after applying a mild trypsin digestion, identifying 40 different proteins, 11 of them identified in our study. Another study reported the proteome of ESP from 21-day-old immature parasites that had been removed from the liver of infected mice and maintained in culture (Robinson et al., 2009). More recently, the changes in the protein profiles of both the NEJ and the host intestinal epithelial cells during the early phase of fasciolosis have been described, analyzing the somatic and tegument proteome of the NEJ after the interaction with host intestinal epithelial cells (González-Miguel et al., 2020). However, to our knowledge, the work described here is the first to investigate and identify proteins in the EVs released from these developmental stages of *F. hepatica*.

We have identified different proteins for each developmental stage (23 from eggs/miracidia and 29 from cultured NEJ). Some of the proteins are described here, as part of EVs, for the first time and some others had already found by other authors.

Some of the proteins identified in EVs from early stages of *F. hepatica* are related to the cytoskeleton, and have been also described previously in somatic and secreted products from *F. hepatica* adults (Morphew et al., 2007), and immature flukes (Robinson et al., 2009), as well as in EVs of *F. hepatica* adults (Cwiklinski et al., 2015). These proteins include different isoforms of actin and tubulins (in miracidia and NEJs), and tropomyosin and dynein in immature flukes, which reflect the active locomotion mechanism displayed by the NEJs, described as a smooth sequence of whole-body waves (González-Miguel et al., 2021).

As has been observed in previous studies, we identified several glycolytic and gluconeogenesis enzymes, as well as molecules related to ATP generation. In EVs from miracidia, phosphoenolpyruvate carboxykinase (PEPCK), glutathione S-transferase, and glyceraldehyde-3-phosphate dehydrogenase (GAPDH) have been identified. PEPCK was established as one of the reliable nuclear markers for species identification of *Fasciola* spp. (Thang et al., 2019), and it has been proposed as an egg antigen in *S. mansoni* (Asahi et al., 2000). Our group also identified PEPCK as an immunodominant antigen recognized by sera from *F. hepatica* infected patients (Marcilla et al., 2008). The PEPCK of *Mycobacterium tuberculosis* also induces strong cell-mediated immune responses in mice (Liu et al., 2006). The glycolytic enzymes identified in NEJ-derived EVs (GAPDH, enolase, pyruvate kinase and TIM), have been reported previously in NEJ or immature fluke secretions (Robinson et al., 2009), or in preparations derived from the NEJ surface (Hernández-González et al., 2010), as well as in EV form *F. hepatica* adults (Marcilla et al., 2012; Cwiklinski et al., 2015). The presence of parasite molecules such as enolase, thioredoxins and glutathione S-transferase as major components of the parasite secretome has been attributed to moonlighting functions usually related with pathogenicity (Bernal et al., 2004; González-Miguel et al., 2019). It has been hypothesized that these proteins aid in the defense against the oxidative burst characteristic of the response of the innate immune cells in response to pathogens. They are also thought to play a role in regulating inflammation and/or in the modulation of host immune responses (Dorey et al., 2021). We hypothesize that some of these molecules could enable parasites to invade and colonize the host tissues.

Other proteins found in *F. hepatica* EVs from NEJs are members of the 14-3-3 family, which have been found on the outer structures, including the oral sucker and on surface of the adult parasites (Robinson et al., 2009; Hernández-González et al., 2010; De la Torre Escudero et al., 2011). Given their possible role in proliferation, migration and changes in morphology, parasite 14-3-3 proteins may be also important during the early stages of *F. hepatica* infection (Siles-Lucas and Gottstein, 2003).

EVs isolated from helminths have been shown to contain a varied array of transmembrane proteins, including some with enzymatic activity (Cwiklinski et al., 2015; Kifle et al., 2020). Supporting this notion, we have identified proteins involved in vesicle biogenesis and trafficking, such as annexin and three tetraspanins in egg/miracidium-derived EVs. In NEJ-derived EVs, other vesicle biogenesis proteins, such as Ras related proteins, including Ras-GEF domain containing protein, small GTP-binding protein Rab1, and ADP-ribosylation factor (Arf) were identified. Proteins from the Ras family are highly represented in EVs from helminths, including EVs released by *F. hepatica* adults (Cwiklinski et al., 2015; Montaño et al., 2021), which make them potential helminth EV biomarker candidates. The RAS superfamily of small GTPases function by initializing signalling cascades involved in various cellular processes, including transcriptional regulation, cell growth, differentiation and regulation of vesicle budding (de Sá-Freire et al., 2003; Wennerberg et al., 2005). Another member of the Ras family present in the EVs released by *F. hepatica* NEJs in culture is the GTP-binding molecule Rab1. A large number of Rab proteins have been identified in *F. hepatica* NEJs (González-Miguel et al., 2020), which are probable key components of the parasite intracellular vesicular transport system, as reported in other eukaryotic cells (Hutagalung and Novick, 2011). Rab proteins are thought to act as molecular “switches” regulating several aspects of vesicle function, such as their formation, transport, tethering, and fusion by interacting with different effector proteins that select cargo, ensure the proper site of fusion and secreting, and promote vesicle movement. This family of proteins have also been shown to regulate both inflammation and innate immunity through the modulation of the formation and fusion of intracellular organelles and their transport (Prashar et al., 2017). Arf proteins function as regulators of actin remodeling and vesicular traffic. Among the functions ascribed to this family of proteins is the activation of phospholipid modifying enzymes, such as phospholipase D, the formation of coated vesicles in the secretory and endocytic pathways, and the maintenance of organelle structure (Béraud-Dufour and Balch, 2001; Chiang et al., 2017).

The transitional endoplasmic reticulum ATPase has been found in NEJ-derived EVs. In rat liver, this ATPase is obligatorily involved in the budding of transition vesicles from the ER, which are thought to be involved in the trafficking of newly synthesized proteins and lipids travelling from the ER to the Golgi (Moreau et al., 1991; Zhang et al., 1994). Their identification in our study suggests that they may have a similar role in the trafficking of proteins and lipids to EVs.

An interesting protein identified in NEJ-EVs is a SEA domain-containing protein, which has been characterized previously in the adult teguments and gastrodermis of *S. japonicum*. This family of proteins are high affinity interactors of glycosaminoglycans and so may be involved in ligand acquisition and immune evasion mechanisms. Moreover, a role as receptors for heme uptake in this hematophagous parasite has been suggested (Mbanefo et al., 2014).

Intriguingly, the cathepsin cysteine peptidases commonly identified in most of *F. hepatica* proteomic studies, have not been identified either in egg/miracidium or NEJ EVs in our study. The most numerous cathepsin L cysteine protease family described to date is that of *F. hepatica*. Most of cathepsins have been described as components of the surface or secretion of NEJs (Hernández-González et al., 2010), and some of the cathepsins have been specifically found in NEJs, but not in adult worms (Cancela et al., 2008). During *F. hepatica* development, cysteine protease production is highly regulated and it has been shown to correlate with the migration and maturation of the parasite. As described, during the *F. hepatica* life cycle, the parasite moves between host organ systems and tissues and so the secretion of proteases and proteins related to protein degradation is likely to be very relevant for this complex life cycle. For instance, in the group of proteolysis-related molecules, we have detected calpain (in egg/miracidium-derived EVs), belonging to the family of calcium-dependent, non-lysosomal cysteine proteases, as well as a peptidase S1 domain-containing protein identified in NEJ-EVs, and ubiquitin and a CUB domain-containing putative protease identified in both, miracidia and NEJs. Secretions from both NEJs and immature flukes have been reported to contain ubiquitin (Robinson et al., 2009). The role of ubiquitination in protein degradation is well known. However, depending on the topology of ubiquitin chains, this post-translational modification can be involved in non-degradative events including trafficking and vesicle fusion events (Mendes et al., 2020). Many CUB domain-containing proteins are proteases. However, CUB domains are widely occurring structural motifs involved in endocytosis, developmental patterning, inflammation and tissue repair and the activation of complement (Blanc et al., 2007).

Penetration of the host intestinal wall by NEJs and the migration of immature flukes through host liver is facilitated by the secretion of different cathepsins and has been suggested to correspond with rapid growth and development of the parasite (reviewed by Robinson et al., 2008). However, histological and immunological studies have shown that NEJs easily penetrate the intestinal wall. Immunolocalization studies of *F. hepatica* NEJ carried out by Cwiklinski et al. (2018) suggest the existence of a reservoir of cathepsin-rich proteolytic capacity in the gastrodermis of the bifurcated gut based on their identification of this class of proteases in the somatic extracts of the parasite. Some of the proteases in the somatic extracts of NEJs were found to be zymogens, suggesting the presence of inactive pre-proteases in secreted granules within the gut epithelium of the parasite (Hernández-González et al., 2010), and not within EVs, justifying their absence in the EVs secreted by the immature parasite. We cannot discard that these proteases are present in NEJ-derived EVs, but if they are present, they are not abundant enough to be detected in our study. In addition, González-Miguel et al. (2020) have suggested that the secretion of certain proteases, such as cathepsins, may be stimulated by the interaction of NEJs with host intestinal epithelial cells. Our data are in accordance with studies showing the secretion of cathepsins in granules from the parasite’s gut epithelium, and with the possible adaptation of NEJ to the host environment since, in our experiments, the EVs have been obtained from NEJs cultured in synthetic media. In this context, the cargo and secretion of EVs may depend on the interaction with the microenvironment and with specific host cells.

In summary, the molecules identified in our proteomic study reflect the intense activity of NEJs at this early stage with several proteins involved in the locomotion process, membrane traffic and cell physiology. This new set of identified proteins in EVs from NEJs and eggs/miracidia of *F. hepatica*, together with those described in NEJ secretions, could help to understand key metabolic, biochemical and molecular mechanisms mediated by EVs that take place after parasite excystment. *F. hepatica* juveniles are thought to have evolved migration mechanisms that serve to evade the host immune system. As the parasite comes in contact with different tissues during the early infection and the ensuing migration that takes place during its life cycle, the success of the infection by *F. hepatica* NEJs depends on rapid metabolic and physiological adjustments to the varying environments and on the evasion of host innate immune cells that are activated by parasite molecules and EVs.

The protein cargo of the *Fh*EVs identified in our study will help to understand the biology of the parasite and the potential interaction of the EVs with the host immune system to prevent parasite expulsion and, in this way, facilitate the infection by *F. hepatica*. Our study could provide, at the diagnostic level, new early potential markers to detect the presence of the parasites in their initial developmental stages and for the design of vaccines or antiparasitic drugs.

## Data Availability Statement

The datasets presented in this study can be found in online repositories. The name of the repository and accession number can be found below: ProteomeXchange Consortium *via* the PRIDE database repository; PXD033086.

## Author Contributions

MT, AM and DB participated in the study concept and design. MT, CS-L, LS-P and DB performed the experiments. VR-T provided the parasites. DB, CS-L and AM analyzed data. DB wrote the manuscript. CS-L and DB prepared the figures. MT, CS-L and AM critically revised the manuscript. All authors contributed to the article and approved the submitted version.

## Funding

This work was supported by the Agencia Estatal de Investigación, Ministerio de Ciencia e Innovación, Spain (Grant number PID2019-105713GB-I00/AEI/10.13039/501100011033), and Conselleria d’Educació, Cultura i Esports, Generalitat Valenciana, Valencia, Spain (Grant PROMETEO/2020/071). CS-L is the recipient of a predoctoral fellowship (PRE2020-092458) funded by AEI/10.13039/501100011033. Part of “Red Traslacional para la Aplicación Clínica de Vesículas Extracelulares, Tentacles”, (RED2018-102411-T, Agencia Estatal de Investigación, Spain).

## Conflict of Interest

The authors declare that the research was conducted in the absence of any commercial or financial relationships that could be construed as a potential conflict of interest.

## Publisher’s Note

All claims expressed in this article are solely those of the authors and do not necessarily represent those of their affiliated organizations, or those of the publisher, the editors and the reviewers. Any product that may be evaluated in this article, or claim that may be made by its manufacturer, is not guaranteed or endorsed by the publisher.
